# Supplementary material for: BTS: a scalable Bayesian Tissue Score for prioritizing GWAS variants and their functional contexts across >1000s of omics datasets
Source: Bioinformatics. 2025 Sep 26;41(10):btaf509. doi: 10.1093/bioinformatics/btaf509 (PMC12517339; doi:10.1093/bioinformatics/btaf509)
Supplement: btaf509_Supplementary_Data [file btaf509_supplementary_data.zip › BTS_supplementary_bioinf_revised.pdf]

Supplementary Information

BTS: scalable Bayesian Tissue Score for prioritizing GWAS variants and their functional contexts across omics data

Pavel P. Kuksa[1,†], Matei Ionita[1,†], Luke Carter[1], Jeffrey Cifello[1], Kaylyn Clark[1], Otto Valladares[1], Yuk Yee Leung[1,*], Li-San Wang[1,*]

[1] Penn Neurodegeneration Genomics Center, Department of Pathology and Laboratory Medicine, University of Pennsylvania, Philadelphia, PA 19104, USA

† The authors wish it to be known that, in their opinions, the first two authors should be regarded as Joint First Authors

* To whom correspondence should be addressed

Email: yyee@pennmedicine.upenn.edu
Email: lswang@pennmedicine.upenn.edu

Supplementary Tables

Supplementary Table S1. Comparison of BTS and other methods.

Supplementary Table S2. Functional genomic and annotation data used for GWAS evaluation with BTS.

Supplementary Table S3. Summary of BTS results for evaluated GWAS datasets

Supplementary Table S4. Metadata for all FILER annotation tracks used in BTS analyses.

Supplementary Table S5. Prioritized annotation tracks, genomic regions, and variants for (a) CAD, (b) IBD, (c) SLE, (d) RA.

Supplementary Table S6. Summary of LD mismatches for CAD GWAS across four 1000 Genomes reference genotype panels.

Supplementary Table S7. Additional supporting evidence for the two prioritized variants (rs9457927, rs9295128) in SLCA22A2 locus.

Supplementary Table S8. Summary of the additional functional genomic annotations in the blood vessel category supporting the BTS-prioritized variants in CAD GWAS.

Supplementary Table S1. Comparison of BTS and other methods.

	Pipeline Features				Pipeline Outputs			
**Method**	**GWAS summary statistics pipeline**	**scalable across many annotation tracks**	**exhaustive causal variant search**	**broad cell type, tissue survey**	**context mapping**	**context-specific variant fine-mapping**	**context-specific locus prioritization**	**References**
INFERNO	yes	yes	no	yes, expandable	partial (annotation overlaps)	no	no	(Kuksa et al., 2020)
FUMA	yes	web-based	no	limited, non-expandable	partial (annotation overlaps)	no	no	(Watanabe et al., 2017)
FINEMAP	no	no	no	no	no	no	no	(Benner et al., 2016)
SuSiE	no	no	no	no	no	no	no	(Wang et al., 2020)
PAINTOR	no	no	yes	no	only small number of tracks	only small number of tracks	no	(Kichaev et al., 2014)
PolyFun	no (only partial)	yes	yes (via PAINTOR)	no	no	yes (via PAINTOR)	no	(Weissbrod et al., 2020)
CARMA	no	yes	no	no	no	yes	no	(Yang et al., 2023)
BTS	yes	yes	yes	yes, expandable	yes	yes	yes	this paper

Supplementary Table S2. Functional genomic and annotation data used for GWAS evaluation with BTS. All annotation tracks were obtained from FILER (Kuksa et al., 2022). Data source column indicates original data source. See **Supplementary Table S4** for full track information.

**Genomic feature type**	**Data source**	**#annotation tracks**	**#cell types**	**#tissue categories**	**#annotated genomic regions**
Enhancers	Roadmap – ChromHMM (Roadmap Epigenomics et al., 2015)	78	65	15	7,877,498
	EpiMap (Boix et al., 2021)	316	110	22	38,053,041
	FANTOM5 (Andersson et al., 2014)	107	77	28	185,397
Open chromatin regions	ENCODE (Consortium, 2012)	203	55	19	33,463,914
	Roadmap (Roadmap Epigenomics et al., 2015)	63	22	7	24,506,793
Active histone marks	ENCODE (Consortium, 2012)	176	47	20	10,956,718
	**TOTAL**	943	194	28	115,043,361

Supplementary Table S3. Summary of BTS results for evaluated GWAS datasets

		CAD (van der Harst & Verweij, 2018)	IBD (Liu et al., 2015)	SLE (Bentham et al., 2015)	RA (Stahl et al., 2010)
GWAS	GWAS variants	7,917,998	10,589,089	7,910,110	2,525,781
Regions of Interest	genome-wide significant variants	4,298	11,967	15,980	3,835
	tag variants	389	885	1,520	753
	regions of interest	167	160	147	58
	variants per region	148	236	200	91
Prioritization	annotations	26	22	11	5
	regions	68	52	44	23
	variants	84	63	44	27
	variant-annotation pairs	1,866	651	97	53
	with increased LL	427	372	41	11

Supplementary Table S4. Metadata for all FILER annotation tracks used in BTS analyses. See **Supplementary_Tables.xlsx.**

Supplementary Table S5. Prioritized annotation tracks, genomic regions, and variants for (a) CAD, (b) IBD, (c) SLE, (d) RA. See **Supplementary_Tables.xlsx.**

Supplementary Table S6. Summary of LD mismatches for CAD GWAS across four 1000 Genomes reference genotype panels. In cases of LD mismatches, BTS consistently prioritizes variants with stronger CAD associations (higher Z-scores).

LD panel	#LD mismatches (LD > 0.9 and |Z-score| difference > 2)	#correct prioritizations by BTS (variant with higher Z-score is prioritized)
EUR	2	2
AFR	4	4
AMR	10	10
EAS	50	50

Supplementary Table S7. Additional supporting evidence for the two prioritized variants (rs9457927, rs9295128) in SLCA22A2 locus (**Figure 4**). Variants were overlapped with the FILER (Kuksa et al., 2022) chromatin interaction, QTL, histone and open chromatin annotation datasets in the blood vessel and blood tissue categories. These genomic features are sourced from ENCODE (Encode Project Consortium, 2012; Encode Project Consortium et al., 2020), ROADMAP (Roadmap Epigenomics Consortium et al., 2015), TF-footprint-atlas (Funk et al., 2020), EpiMap (Boix et al., 2021), GTEx version 8 (GTEx Consortium, 2020), eQTL Catalogue release 6 (Kerimov et al., 2021), 3D Genome (Wang et al., 2018), and pcHiC (Javierre et al., 2016; Jung et al., 2019).

**Genomic feature**	**Tissue category**	**Data source**
**rs9457927**		
Open chromatin (DNase-seq)	Blood Vessel	TF-footprint-atlas
Hi-C chromatin interactions	Blood	ENCODE
eQTL SNP significant associations	Blood	GTEx_v8
eQTL SNP significant associations	Blood	eQTL_Catalogue
pcHi-C chromatin interactions	Blood	Javierre_2016_pcHiC
pcHi-C chromatin interactions	Blood Vessel	Javierre_2016_pcHiC
**rs9295128**		
Open chromatin (ATAC-seq)	Blood	ENCODE
Enhancer	Blood Vessel	EpiMap_Enhancers
Hi-C chromatin interactions	Blood Vessel	3DGenome
Histone ChIP-seq H3K27ac	Blood Vessel	ENCODE
Histone ChIP-seq H3K27ac	Blood Vessel	ROADMAP
Histone ChIP-seq H3K4me1	Blood	ENCODE
Histone ChIP-seq H3K4me1	Blood Vessel	ENCODE
Histone ChIP-seq H3K4me1	Blood Vessel	ROADMAP
eQTL SNP significant associations	Blood	GTEx_v8
eQTL SNP significant associations	Blood	eQTL_Catalogue

pcHi-C chromatin interactions	Blood Vessel	Jung_2019_pcHiC

Supplementary Table S8. Summary of the additional functional genomic annotations in the blood vessel category supporting the BTS-prioritized variants in CAD GWAS. BTS-prioritized variants were compared to FG annotations from the FILER database (Kuksa et al., 2022), and the overlaps in blood vessel category were collected and summarized. These supporting data are sourced ENCODE (Encode Project Consortium, 2012; Encode Project Consortium et al., 2020), ROADMAP (Roadmap Epigenomics Consortium et al., 2015), TF-footprint-atlas (Funk et al., 2020), EpiMap (Boix et al., 2021), GTEx version 8 (GTEx Consortium, 2020), eQTL Catalogue release 6 (Kerimov et al., 2021), 3D Genome (Wang et al., 2018), pcHiC (Javierre et al., 2016; Jung et al., 2019), and ABC enhancer-gene interactions (Nasser et al., 2021).

Genomic data type	Supporting data source(s)	Overlapping BTS-prioritized CAD variants
Open chromatin (ATAC-seq)	ENCODE	18 (rs11616893,rs11619113,rs11670056,rs1169288,rs12740374,rs1412444,rs1446668,rs146534110,rs17242395,rs17293632,rs2891168,rs35932524,rs3783320,rs604723,rs6511720,rs7296737,rs9337951,rs998584)
Open chromatin (DNase-seq)	TF footprint atlas	12 (rs11670056,rs12740374,rs1446668,rs17293632,rs2107595,rs35932524,rs3783320,rs604723,rs61075980,rs7296737,rs7500448,rs998584)
Enhancer	EpiMap enhancers, ROADMAP enhancers, ABC enhancer-gene interactions	33 (rs10899953,rs11616893,rs11619113,rs11635498,rs11670056,rs1169288,rs12522841,rs12740374,rs1377197,rs1412444,rs146534110,rs17242395,rs17293632,rs1869204,rs2074633,rs2107595,rs2891168,rs35932524,rs3741380,rs3783320,rs3803809,rs4948792,rs604723,rs61075980,rs6511720,rs67703045,rs72749233,rs7296737,rs7500448,rs78030362,rs9337951,rs944172,rs998584)
Hi-C chromatin interactions	3DGenome	6 (rs1966248,rs28601761,rs4543559,rs644045,rs7502499,rs78030362)
Histone ChIP-seq H3K27ac	ENCODE, ROADMAP	29 (rs11072813,rs11616893,rs11619113,rs11635498,rs11670056,rs1169288,rs1412444,rs1446668,rs146534110,rs17163363,rs17242395,rs17293632,rs2107595,rs35932524,rs3741380,rs3783320,rs3803809,rs5015801,rs604723,rs6511720,rs67703045,rs6905288,rs72749233,rs7296737,rs7500448,rs78030362,rs9337951,rs944172,rs998584)

Histone ChIP-seq H3K4me1	ENCODE, ROADMAP	32 (rs11072813,rs11616893,rs11619113,rs11635498,rs11670056,rs1169288,rs117448071,rs12522841,rs12740374,rs1446668,rs146534110,rs17163363,rs17242395,rs17293632,rs2107595,rs35932524,rs3741380,rs3783320,rs3803809,rs4678145,rs4948792,rs604723,rs6511720,rs67703045,rs72749233,rs7296737,rs7500448,rs7502499,rs78030362,rs9337951,rs944172,rs998584)
Histone ChIP-seq H3K4me3	ENCODE, ROADMAP	20 (rs11072813,rs11616893,rs11619113,rs11635498,rs11670056,rs1169288,rs1446668,rs17163363,rs17242395,rs17293632,rs2107595,rs2128739,rs35932524,rs3783320,rs4678145,rs604723,rs6511720,rs72749233,rs7500448,rs9337951)
TF ChIP-seq	ENCODE	2 (rs1446668,rs3783320)
eQTL SNP significant associations	GTEx v8, eQTL Catalogue	29 (rs10083700,rs11072813,rs11635498,rs11670056,rs1169288,rs12127701,rs13134800,rs1412444,rs1446668,rs17163363,rs17242395,rs17463531,rs2074633,rs2107595,rs2128739,rs35932524,rs3741380,rs3783320,rs4678145,rs604723,rs644045,rs72749233,rs7500448,rs7502499,rs8003602,rs9337951,rs9381462,rs9457827,rs9979847)
pcHi-C chromatin interactions	pcHiC (Javierre et al 2016), pcHiC (Jung et al 2019)	38 (rs10083700,rs10899953,rs11635498,rs11670056,rs1169288,rs117448071,rs12127701,rs12740374,rs1412444,rs141622900,rs1446668,rs17163363,rs17242395,rs17293632,rs17463531,rs1869204,rs1966248,rs2107595,rs28601761,rs2891168,rs3741380,rs3783320,rs3803809,rs4543559,rs4678145,rs4948792,rs5015801,rs55682375,rs604723,rs644045,rs6511720,rs67703045,rs7502499,rs78030362,rs8003602,rs9337951,rs9457827,rs9979847)
sQTL SNP significant associations	eQTL Catalogue, GTEx v8	11 (rs10083700,rs11670056,rs1169288,rs12127701,rs1412444,rs1446668,rs3741380,rs644045,rs6511720,rs7296737,rs9381462)

Supplementary Figures

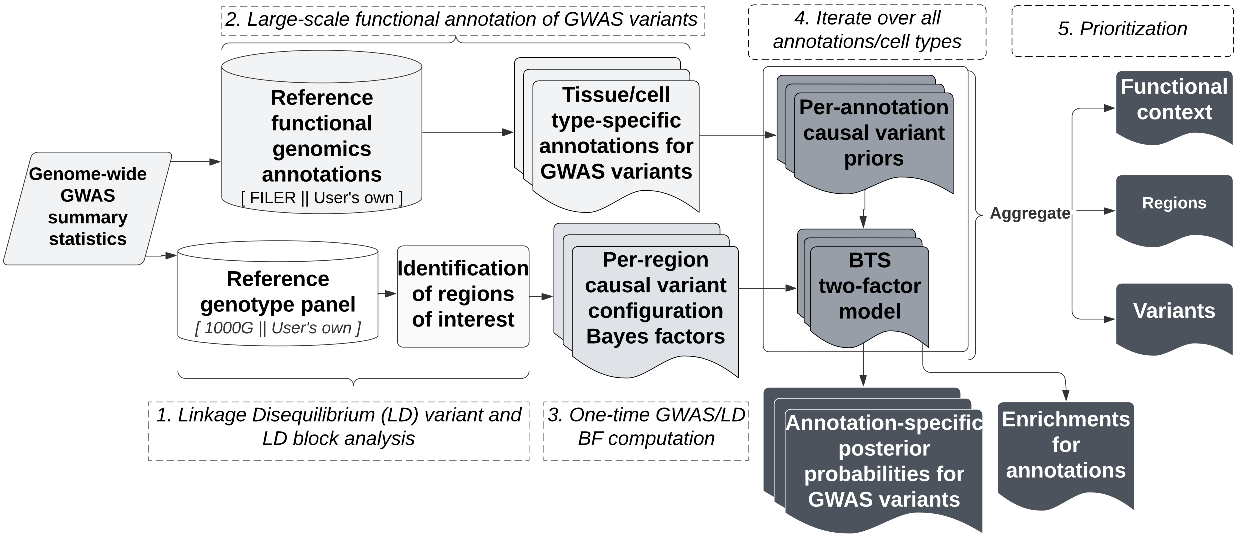

Supplementary Figure S1. Overview of BTS GWAS summary statistics analysis pipeline. Input GWAS summary statistics are first analyzed to determine a set of genomic regions for further functional analysis and fine-mapping (Step 1; **Section** "BTS GWAS summary statistics analysis workflow"**; Methods; Supplementary Methods**). Then, for each of identified genomic regions all the corresponding GWAS variants are annotated with genome-wide tissue and cell type-specific annotations of interest (Step 2; Section "BTS GWAS summary statistics analysis workflow"; **Supplementary Methods**). Computed per-locus variant LD correlation matrices, variant GWAS Z-scores, and variant functional annotations (Steps 1,2) are used as input to BTS model (Section "BTS statistical model"). BTS first pre-computes Bayesian Factors for all possible causal variant configurations across all loci (Step 3; **Section** "BTS statistical model") and is then applied iteratively (Step 4; **Section** "BTS GWAS summary statistics analysis workflow") for each of the provided functional annotations. BTS outputs annotation relevance (enrichments), locus-level probabilities, and context-specific causal variant posterior probabilities. The two-factor structure of BTS model (Steps 3, 4; Section "BTS statistical model") allows it to be evaluated very efficiently across thousands of genome-wide annotations (**Fig. 6; Section** "Running time improvement"; **Methods**). Each of the provided annotations, identified genomic regions, and GWAS variants within these regions are then prioritized by BTS based on their estimated enrichments (prior odds), observed increase in causal posterior likelihood, and variant posteriors, respectively (Step 5; **Fig. 3**; **Sections "**Prioritizing regions, variants and their contexts with BTS**", "**Cross-trait BTS evaluation**"**).

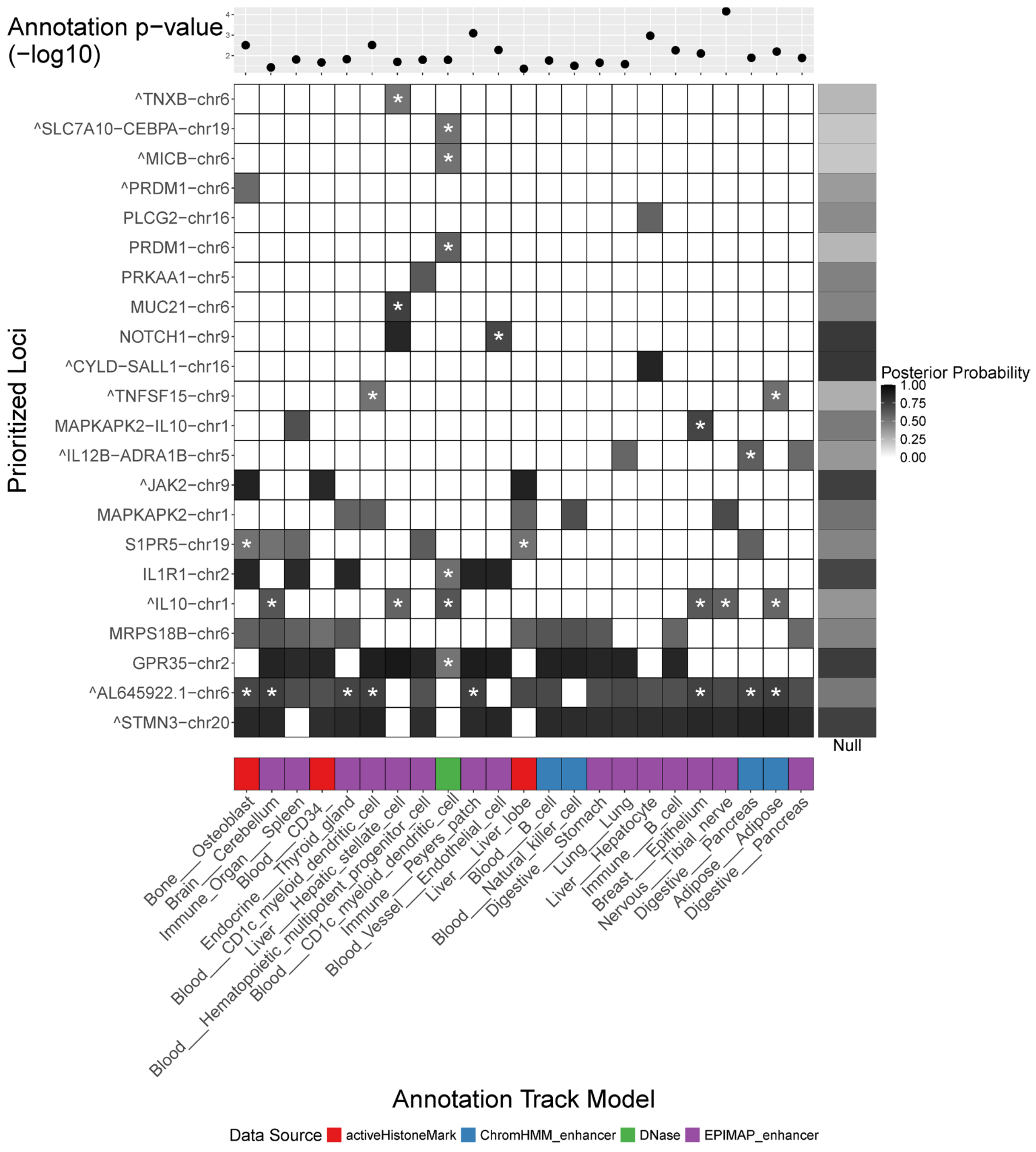

Supplementary Figure S2. BTS prioritizes digestive, blood, and immune tissues and annotations based on IBD GWAS summary statistics. Shown are prioritized genomic regions (Y axis) and their functional contexts (X axis). For each region and context shown is the top variant posterior (shades of gray) with the star (*) indicating posterior increase of at least .2 in that context compared to the null model without annotation (right).

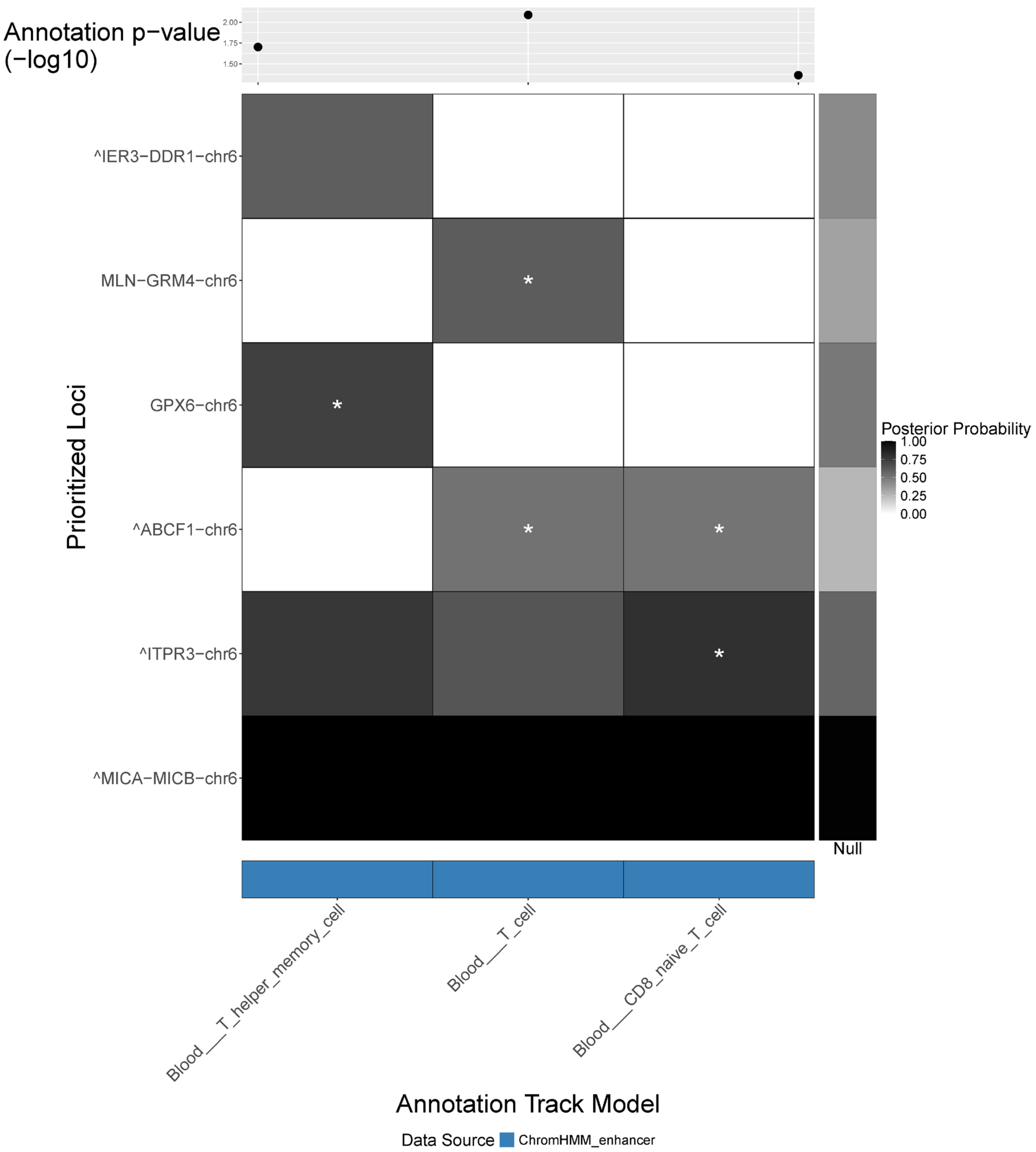

Supplementary Figure S3. BTS prioritizes T cells and ChromHMM enhancer annotations for RA. Shown are prioritized genomic regions (Y axis) and their functional contexts (X axis). For each region and context shown is the top variant posterior (shades of gray) with the star (*) indicating posterior increase of at least .2 in that context compared to the null model without annotation (right).

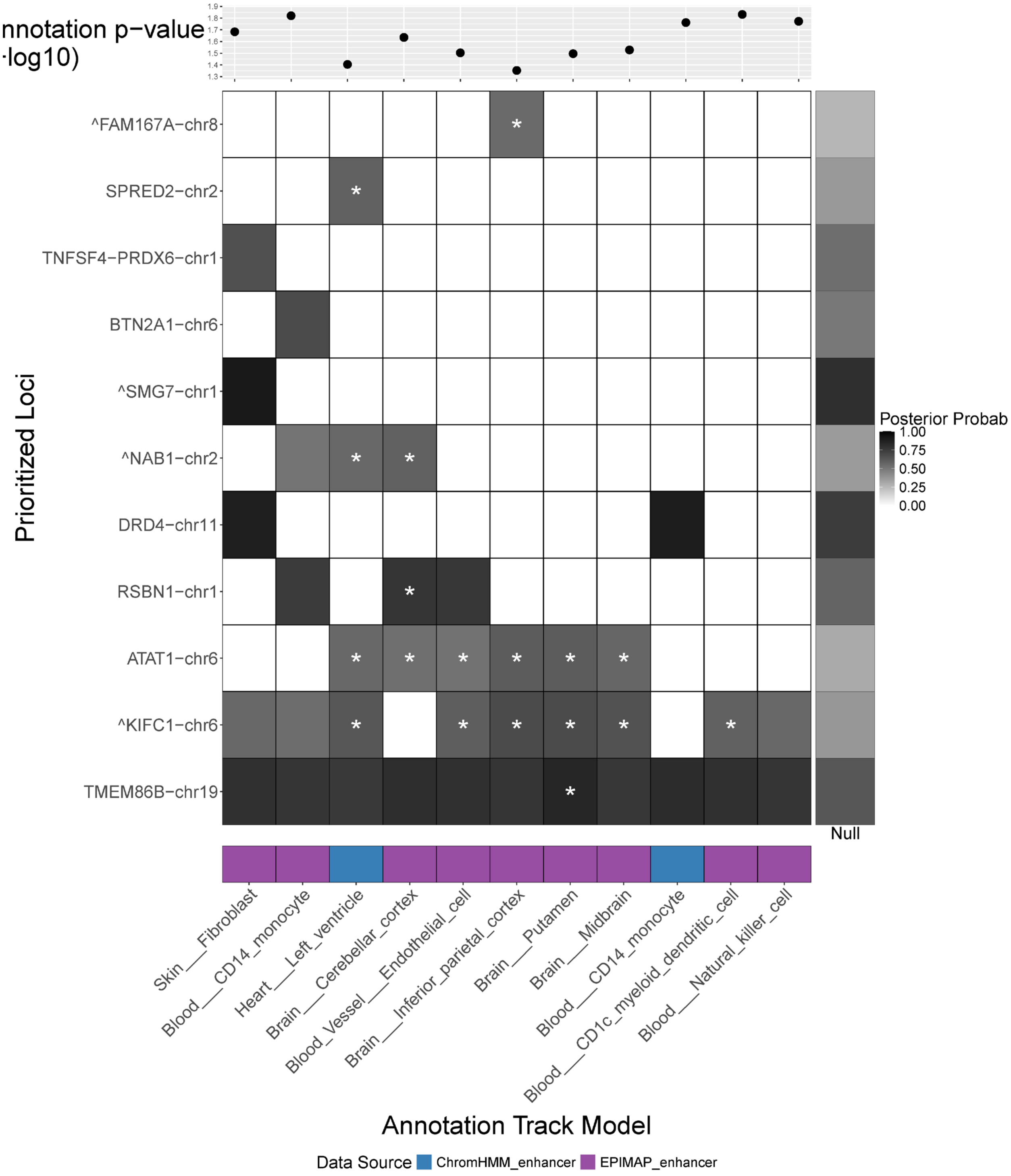

Supplementary Figure S4. BTS prioritizes heart left ventricle, monocyte, and brain tissues and annotations based on SLE GWAS summary statistics. Shown are prioritized genomic regions (Y axis) and their functional contexts (X axis). For each region and context shown is the top variant posterior (shades of gray) with the star (*) indicating posterior increase of at least .2 in that context compared to the null model without annotation (right).

Immune-related category prioritized by BTS.

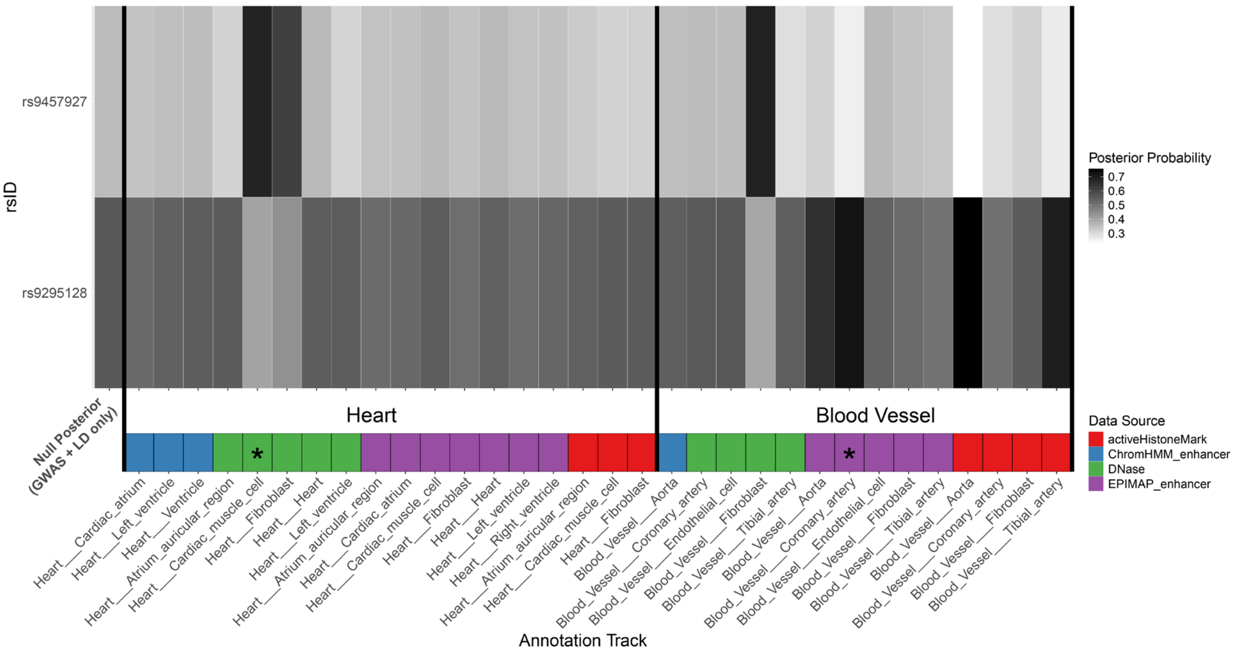

Supplementary Figure S5. Differential cell-type and tissue-specific credible set variant prioritization for SLCA22A region. Shown are variant posteriors across heart and blood vessel annotations. Also shown are the baseline variant posteriors (left) for model without any annotations (estimated using GWAS Z-scores and LD information only). Variants rs9295128 and rs9457927 are prioritized in different cell and genomic feature contexts including coronary artery (EpiMap enhancer) and cardiac muscle cell open chromatin (DNase) contexts, respectively. Annotation tracks displayed in **Figure 4** are marked with a star (*).

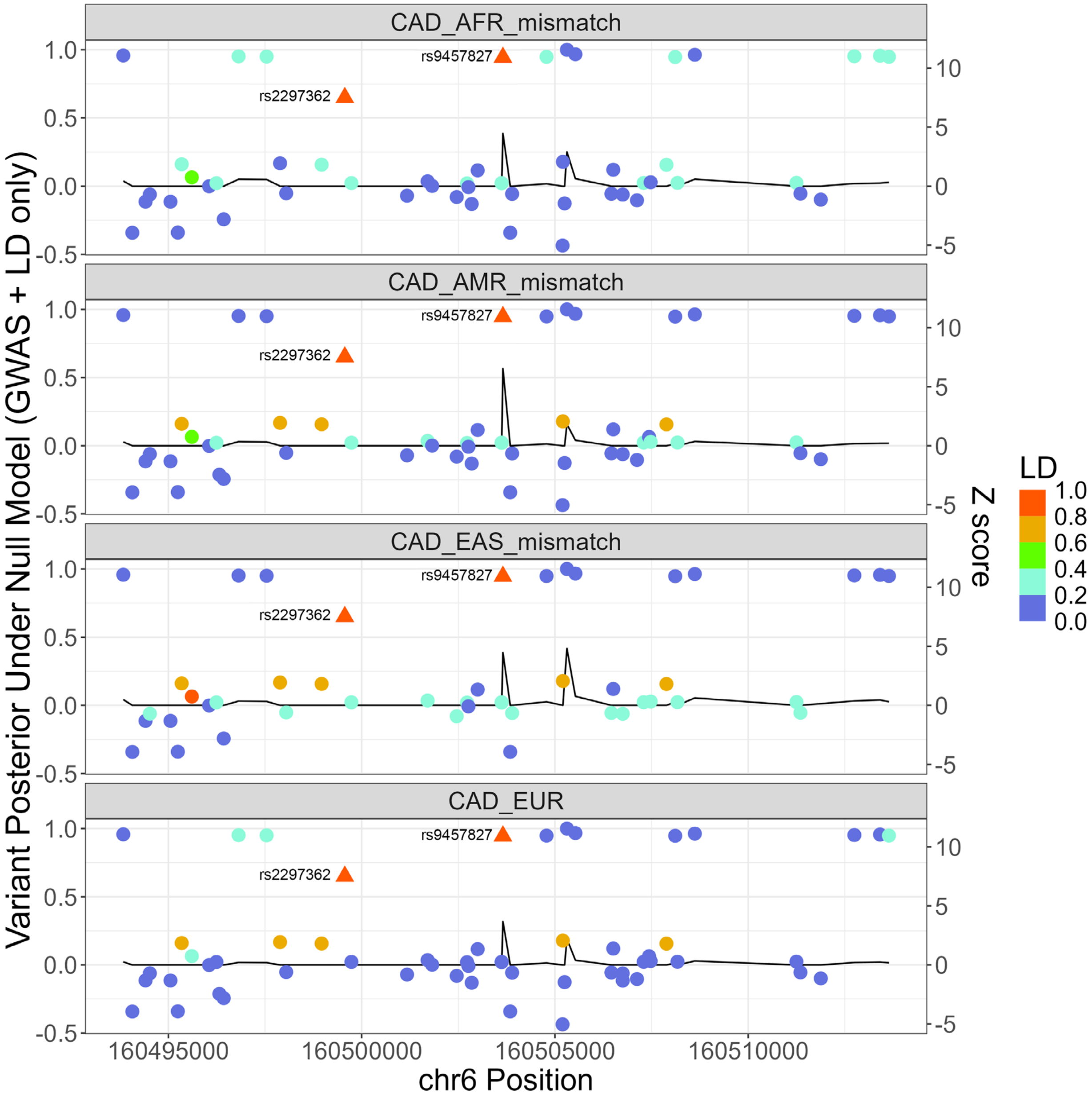

Supplementary Figure S6. An example of variant prioritization in the cases of LD mismatches for CAD GWAS IGFR2 locus across multiple ancestries. The original GWAS uses European (EUR) genotype panels from the 1000 Genomes project (Genomes Project Consortium et al., 2015), which is compared to other 1000 Genomes genotype panels including African (AFR), Admixed American (AMR), and East Asian (EAS). Between the two variants in strong LD (rs2297362, rs9457827), BTS prioritizes the variant with higher GWAS Z-score (rs9457827) across all genotype reference panels (black line), including those with LD mismatch.

Supplementary References

Andersson, R., Gebhard, C., Miguel-Escalada, I., Hoof, I., Bornholdt, J., Boyd, M., Chen, Y., Zhao, X., Schmidl, C., Suzuki, T., Ntini, E., Arner, E., Valen, E., Li, K., Schwarzfischer, L., Glatz, D., Raithel, J., Lilje, B., Rapin, N., . . . Sandelin, A. (2014). An atlas of active enhancers across human cell types and tissues. *Nature*, *507*(7493), 455-461. https://doi.org/10.1038/nature12787

Benner, C., Spencer, C. C., Havulinna, A. S., Salomaa, V., Ripatti, S., & Pirinen, M. (2016). FINEMAP: efficient variable selection using summary data from genome-wide association studies. *Bioinformatics*, *32*(10), 1493-1501. https://doi.org/10.1093/bioinformatics/btw018

Bentham, J., Morris, D. L., Graham, D. S. C., Pinder, C. L., Tombleson, P., Behrens, T. W., Martin, J., Fairfax, B. P., Knight, J. C., Chen, L., Replogle, J., Syvanen, A. C., Ronnblom, L., Graham, R. R., Wither, J. E., Rioux, J. D., Alarcon-Riquelme, M. E., & Vyse, T. J. (2015). Genetic association analyses implicate aberrant regulation of innate and adaptive immunity genes in the pathogenesis of systemic lupus erythematosus. *Nat Genet*, *47*(12), 1457-1464. https://doi.org/10.1038/ng.3434

Boix, C. A., James, B. T., Park, Y. P., Meuleman, W., & Kellis, M. (2021). Regulatory genomic circuitry of human disease loci by integrative epigenomics. *Nature*, *590*(7845), 300-307. https://doi.org/10.1038/s41586-020-03145-z

Encode Project Consortium. (2012). An integrated encyclopedia of DNA elements in the human genome. *Nature*, *489*(7414), 57-74. https://doi.org/10.1038/nature11247

Encode Project Consortium, Moore, J. E., Purcaro, M. J., Pratt, H. E., Epstein, C. B., Shoresh, N., Adrian, J., Kawli, T., Davis, C. A., Dobin, A., Kaul, R., Halow, J., Van Nostrand, E. L., Freese, P., Gorkin, D. U., Shen, Y., He, Y., Mackiewicz, M., Pauli-Behn, F., . . . Weng, Z. (2020). Expanded encyclopaedias of DNA elements in the human and mouse genomes. *Nature*, *583*(7818), 699-710. https://doi.org/10.1038/s41586-020-2493-4

Funk, C. C., Casella, A. M., Jung, S., Richards, M. A., Rodriguez, A., Shannon, P., Donovan-Maiye, R., Heavner, B., Chard, K., Xiao, Y., Glusman, G., Ertekin-Taner, N., Golde, T. E., Toga, A., Hood, L., Van Horn, J. D., Kesselman, C., Foster, I., Madduri, R., . . . Ament, S. A. (2020). Atlas of Transcription Factor Binding Sites from ENCODE DNase Hypersensitivity Data across 27 Tissue Types. *Cell Rep*, *32*(7), 108029. https://doi.org/10.1016/j.celrep.2020.108029

Genomes Project Consortium, Auton, A., Brooks, L. D., Durbin, R. M., Garrison, E. P., Kang, H. M., Korbel, J. O., Marchini, J. L., McCarthy, S., McVean, G. A., & Abecasis, G. R. (2015). A global reference for human genetic variation. *Nature*, *526*(7571), 68-74. https://doi.org/10.1038/nature15393

GTEx Consortium. (2020). The GTEx Consortium atlas of genetic regulatory effects across human tissues. *Science*, *369*(6509), 1318-1330. https://doi.org/10.1126/science.aaz1776

Javierre, B. M., Burren, O. S., Wilder, S. P., Kreuzhuber, R., Hill, S. M., Sewitz, S., Cairns, J., Wingett, S. W., Varnai, C., Thiecke, M. J., Burden, F., Farrow, S., Cutler, A. J., Rehnstrom, K., Downes, K., Grassi, L., Kostadima, M., Freire-Pritchett, P., Wang, F., . . . Fraser, P. (2016). Lineage-Specific Genome Architecture Links Enhancers and Non-coding Disease Variants to Target Gene Promoters. *Cell*, *167*(5), 1369-1384 e1319. https://doi.org/10.1016/j.cell.2016.09.037

Jung, I., Schmitt, A., Diao, Y., Lee, A. J., Liu, T., Yang, D., Tan, C., Eom, J., Chan, M., Chee, S., Chiang, Z., Kim, C., Masliah, E., Barr, C. L., Li, B., Kuan, S., Kim, D., & Ren, B. (2019).

A compendium of promoter-centered long-range chromatin interactions in the human genome. *Nat Genet*, *51*(10), 1442-1449. https://doi.org/10.1038/s41588-019-0494-8

Kerimov, N., Hayhurst, J. D., Peikova, K., Manning, J. R., Walter, P., Kolberg, L., Samovica, M., Sakthivel, M. P., Kuzmin, I., Trevanion, S. J., Burdett, T., Jupp, S., Parkinson, H., Papatheodorou, I., Yates, A. D., Zerbino, D. R., & Alasoo, K. (2021). A compendium of uniformly processed human gene expression and splicing quantitative trait loci. *Nat Genet*, *53*(9), 1290-1299. https://doi.org/10.1038/s41588-021-00924-w

Kichaev, G., Yang, W. Y., Lindstrom, S., Hormozdiari, F., Eskin, E., Price, A. L., Kraft, P., & Pasaniuc, B. (2014). Integrating functional data to prioritize causal variants in statistical fine-mapping studies. *PLoS Genet*, *10*(10), e1004722. https://doi.org/10.1371/journal.pgen.1004722

Kuksa, P. P., Lee, C. Y., Amlie-Wolf, A., Gangadharan, P., Mlynarski, E. E., Chou, Y. F., Lin, H. J., Issen, H., Greenfest-Allen, E., Valladares, O., Leung, Y. Y., & Wang, L. S. (2020). SparkINFERNO: a scalable high-throughput pipeline for inferring molecular mechanisms of non-coding genetic variants. *Bioinformatics*, *36*(12), 3879-3881. https://doi.org/10.1093/bioinformatics/btaa246

Kuksa, P. P., Leung, Y. Y., Gangadharan, P., Katanic, Z., Kleidermacher, L., Amlie-Wolf, A., Lee, C. Y., Qu, L., Greenfest-Allen, E., Valladares, O., & Wang, L. S. (2022). FILER: a framework for harmonizing and querying large-scale functional genomics knowledge. *NAR Genom Bioinform*, *4*(1), lqab123. https://doi.org/10.1093/nargab/lqab123

Liu, J. Z., van Sommeren, S., Huang, H., Ng, S. C., Alberts, R., Takahashi, A., Ripke, S., Lee, J. C., Jostins, L., Shah, T., Abedian, S., Cheon, J. H., Cho, J., Dayani, N. E., Franke, L., Fuyuno, Y., Hart, A., Juyal, R. C., Juyal, G., . . . Weersma, R. K. (2015). Association analyses identify 38 susceptibility loci for inflammatory bowel disease and highlight shared genetic risk across populations. *Nat Genet*, *47*(9), 979-986. https://doi.org/10.1038/ng.3359

Nasser, J., Bergman, D. T., Fulco, C. P., Guckelberger, P., Doughty, B. R., Patwardhan, T. A., Jones, T. R., Nguyen, T. H., Ulirsch, J. C., Lekschas, F., Mualim, K., Natri, H. M., Weeks, E. M., Munson, G., Kane, M., Kang, H. Y., Cui, A., Ray, J. P., Eisenhaure, T. M., . . . Engreitz, J. M. (2021). Genome-wide enhancer maps link risk variants to disease genes. *Nature*, *593*(7858), 238-243. https://doi.org/10.1038/s41586-021-03446-x

Roadmap Epigenomics Consortium, Kundaje, A., Meuleman, W., Ernst, J., Bilenky, M., Yen, A., Heravi-Moussavi, A., Kheradpour, P., Zhang, Z., Wang, J., Ziller, M. J., Amin, V., Whitaker, J. W., Schultz, M. D., Ward, L. D., Sarkar, A., Quon, G., Sandstrom, R. S., Eaton, M. L., . . . Kellis, M. (2015). Integrative analysis of 111 reference human epigenomes. *Nature*, *518*(7539), 317-330. https://doi.org/10.1038/nature14248

Stahl, E. A., Raychaudhuri, S., Remmers, E. F., Xie, G., Eyre, S., Thomson, B. P., Li, Y., Kurreeman, F. A., Zhernakova, A., Hinks, A., Guiducci, C., Chen, R., Alfredsson, L., Amos, C. I., Ardlie, K. G., Consortium, B., Barton, A., Bowes, J., Brouwer, E., . . . Plenge, R. M. (2010). Genome-wide association study meta-analysis identifies seven new rheumatoid arthritis risk loci. *Nat Genet*, *42*(6), 508-514. https://doi.org/10.1038/ng.582

van der Harst, P., & Verweij, N. (2018). Identification of 64 Novel Genetic Loci Provides an Expanded View on the Genetic Architecture of Coronary Artery Disease. *Circ Res*, *122*(3), 433-443. https://doi.org/10.1161/CIRCRESAHA.117.312086

Wang, G., Sarkar, A., Carbonetto, P., & Stephens, M. (2020). A simple new approach to variable selection in regression, with application to genetic fine mapping. *J R Stat Soc Series B Stat Methodol*, *82*(5), 1273-1300. https://doi.org/10.1111/rssb.12388

Wang, Y., Song, F., Zhang, B., Zhang, L., Xu, J., Kuang, D., Li, D., Choudhary, M. N. K., Li, Y., Hu, M., Hardison, R., Wang, T., & Yue, F. (2018). The 3D Genome Browser: a web-

based browser for visualizing 3D genome organization and long-range chromatin interactions. *Genome Biol*, *19*(1), 151. https://doi.org/10.1186/s13059-018-1519-9

Watanabe, K., Taskesen, E., van Bochoven, A., & Posthuma, D. (2017). Functional mapping and annotation of genetic associations with FUMA. *Nat Commun*, *8*(1), 1826. https://doi.org/10.1038/s41467-017-01261-5

Weissbrod, O., Hormozdiari, F., Benner, C., Cui, R., Ulirsch, J., Gazal, S., Schoech, A. P., van de Geijn, B., Reshef, Y., Marquez-Luna, C., O'Connor, L., Pirinen, M., Finucane, H. K., & Price, A. L. (2020). Functionally informed fine-mapping and polygenic localization of complex trait heritability. *Nat Genet*, *52*(12), 1355-1363. https://doi.org/10.1038/s41588-020-00735-5

Yang, Z., Wang, C., Liu, L., Khan, A., Lee, A., Vardarajan, B., Mayeux, R., Kiryluk, K., & Ionita-Laza, I. (2023). CARMA is a new Bayesian model for fine-mapping in genome-wide association meta-analyses. *Nat Genet*, *55*(6), 1057-1065. https://doi.org/10.1038/s41588-023-01392-0

Supplementary Methods

1 BTS GWAS summary statistics analysis workflow

Input

1. Genome-wide GWAS summary statistics dataset G (Z-scores)
2. Reference genotype panel R for LD estimation (e.g., 1000 Genomes EUR super-population) or pre-computed genome-wide pairwise LD scores
3. Reference variant information V [e.g, dbSNP]
4. Reference functional genomic and annotation tracks data collection $D = \{D_1, \ldots, D_K\}$ and corresponding metadata $MD = \{MD_1, \ldots, MD_K\}$ [e.g., FILER]
5. Genomic regions of interest (GWAS loci) $I = \{I_1, \ldots, I_N\}$, where each $I_j = [chr_j, left_j, right_j]$ is a genomic region to be analyzed and fine-mapped. These genomic regions of interest can be either user-supplied or inferred from GWAS summary statistics G and R by GWAS genome-wide signficant variant LD pruning/LD expansion (see **Methods**).

BTS GWAS summary statistics analysis algorithm

1. GWAS1. From G and reference variant information database V, infer reference and non-reference alleles for each reported variant/association and compute non-reference-allele adjusted summary statistics G_{std} and reference/non-reference variant representations [*Giggle, dbSNP*]
2. GWAS2. Using G_{std} and R, obtain regions of interest by first LD pruning (to obtain a set of pairwise independent variants) and then LD-expanding genome-wide significant ($p < 5e-8$) GWAS variants from G_{std} to obtain a set of LD blocks. Merge any overlapping LD blocks to obtain a final set of non-overlapping regions of interest L_j, $j = 1 \ldots N$ for further analysis.
3. GWAS3. From G_{std}, obtain Z-scores for each locus j, $Z_j = \frac{\beta_j^{non-ref}}{se_j}$, $[|L_j| \times 1]$, where $|L_j|$ is the number of variants in locus j.
4. LD. Compute pairwise LD matrix LD_j, $[|L_j| \times |L_j|]$, for every locus j using R [or extract LD_j from the pre-computed LD scores] [*Giggle, plink*]
5. FG. For each locus j, compute variant annotation matrices $A^j_{i,k} = 1, [|L_j| \times K]$ if the ith SNP in locus j overlaps kth annotation $D_k, k = 1 \ldots K$. Note: annotations maybe selected based on the annotation metadata MD. [*FILER*]
6. BTS1. Pre-compute probabilities for all possible causal configuration C_j, $[\sum_{i=0}^{i=d} \binom{|L_j|}{i} \times 1]$ for each locus j using LD_j and Z_j [Bayesian factors $BF_c, c = 1, \ldots, |C_j|$; `compute_configs(LD,Z)`]
7. BTS2. Run EM using the pre-computed configuration probabilities BF_{C_j} and annotation matrix A^j, $j = 1, \ldots, L$ [`run_em(P(C), A)`]
   - estimate annotation enrichment/effect sizes γ_k for every genomic annotation track D_k, $k = 1, \ldots, K$
   - compute variant posteriors $P^j(C_i)$ for every variant $i = 1, \ldots, |L_j|$ and locus $j = 1, \ldots, N$
8. BTS3. Summarize each of the $K + 1$ estimated models across L loci [`get_model_summary`]

- O1. per-annotation variant causal posteriors $P^j_{i,k}$, $j = 1, \ldots, L$, $i = 1, \ldots, |L_j|$,$k = 1, \ldots, K$
- O2. (optimized) total data likelihoods LL_k for each model $k = 1, \ldots, K$
- O3. annotation relevance / enrichments $E_k, k = 1, \ldots, K$
- O4. annotation-specific locus likelihoods ll^{locus}_j and significance scores $p^{locus}_{j,k}$, $j = 1, \ldots, N$, $k = 1, \ldots, K$

Main data objects: Loci $L = \{L_1, ..., L_L\}$, SNPs $S = \{S_1, ..., S_L\}$, summary statistics $Z = \{Z_1, ..., Z_L\}$, locus LD matrices $LD = \{LD_1, ..., LD_L\}$ reference annotation data $D = \{D_1, ..., D_K\}$, metadata $MD = \{MD_1, \ldots, MD_K\}$, variant/loci annotation matrices $\{A^j_{i,k}\}$, $j = 1, \ldots, L$, $i = 1, \ldots, L_j$, $k = 1, \ldots, K$

2 Causal variant configuration modeling

Let Z be a vector of GWAS Z-scores (standardized regression coefficients), Λ a vector of true effect sizes (unknown), Σ the correlation (LD) matrix, and C a binary vector of causal variant status. We want to estimate the probability of each causal configuration C, conditional on the observed data and the model parameters:

$$P(C|Z, \Lambda, \Sigma). \tag{1}$$

We use Bayes' theorem:

$$P(C|Z, \Lambda, \Sigma) = \frac{P(Z|C, \Lambda, \Sigma) \cdot P(C)}{P(Z|\Lambda, \Sigma)}. \tag{2}$$

On the right hand-side, $P(Z|C, \Lambda, \Sigma)$ is the likelihood of observing the data Z under configuration C, $P(C)$ is the prior of the configuration (which will depend on functional annotations of the variants). The denominator is the total data likelihood under the model; to compute it is difficult and unnecessary, because it suffices to consider probability ratios, relative to the null configuration:

$$\frac{P(C|Z, \Lambda, \Sigma)}{P(C = 0|Z, \Lambda, \Sigma)} = \frac{P(Z|C, \Lambda, \Sigma) \cdot P(C)}{P(Z|C = 0, \Lambda, \Sigma) \cdot P(C = 0)} = BF(C, Z, \Sigma)\frac{P(C)}{P(C = 0)}. \tag{3}$$

The Bayes factor is, by definition, the ratio of likelihoods. It will turn out to have a dominant contribution in the posterior probabilities, while the priors act as fine-tuning or tie-breakers. This accurately models our expectation that GWAS summary statistics should carry more weight than functional annotations: if a variant has negligible association, is should never be prioritized.

3 Modeling and computing configuration Bayes factors

We model the data likelihood for summary statistics as a multivariate normal:

$$P(Z|C, \Lambda, \Sigma) = \mathcal{N}(Z; \Sigma \cdot \Lambda \circ C, \Sigma). \tag{4}$$

We also model the effect sizes as normal, with mean 0 and diagonal variance, using the scalar W as a model parameter:

$$\Lambda \sim \mathcal{N}(0, W \cdot I). \tag{5}$$

Combining 4 and 5 gives a multivariate normal with mean 0 and variance $\Sigma + Var(\Sigma \cdot \Lambda \circ C)$. Computing the second term gives:

$$P(Z|C, \Sigma) = \mathcal{N}(Z; 0, \Sigma + W \cdot \Sigma C \Sigma). \tag{6}$$

For the null configuration, $C = 0$, and $P(Z|C, \Sigma) = \mathcal{N}(Z; 0, \Sigma)$.

Permuting labels if necessary, we can assume that causal configuration $C = (C_1|C_0)$, where C_1 consists of ones (the causal variants) and C_0 consists of zeros. Let $Z = (Z_1|Z_0)$ be the decomposition of Z corresponding to causal and non-causal blocks, and similarly for Σ:

$$\Sigma = \left(\begin{array}{c|c} \Sigma_{11} & \Sigma_{10} \\ \hline \Sigma_{01} & \Sigma_{00} \end{array}\right). \tag{7}$$

4 Computing Bayes factors efficiently

Efficient computatio of Bayes factors in BTS is achieved using the following two lemmas/observations.

Lemma 1 allows to speed-up Bayes factor computation by ignoring all the blocks involving non-causal variants, and work with much smaller arrays ([1], [2]).

Lemma 1

$$\frac{\mathcal{N}(Z; 0, \Sigma + W \cdot \Sigma C \Sigma)}{\mathcal{N}(Z; 0, \Sigma)} = \frac{\mathcal{N}(Z_1; 0, \Sigma_{11} + W \cdot \Sigma_{11}^2)}{\mathcal{N}(Z_1; 0, \Sigma_{11})}. \tag{8}$$

Our own observation is that this can be further simplified (**Lemma 2**).

Lemma 2

$$\frac{\mathcal{N}(Z_1; 0, \Sigma_{11} + W \cdot \Sigma_{11}^2)}{\mathcal{N}(Z_1; 0, \Sigma_{11})} = \det(I + W\Sigma_{11})^{-1/2} \exp\left(\frac{W}{2} Z_1^T (I + W\Sigma_{11})^{-1} Z_1\right). \tag{9}$$

The advantages of this formulation are:

- it computes a single matrix inverse instead of two, decreasing computation time;
- the matrix being inverted, $I + W\Sigma_{11}$, is always strictly positive definite, even when Σ_{11} is singular. This helps with numerical precision, and avoids the need for any regularization of Σ_{11}.

To prove Lemma 2, start from the left-hand side, writing out the multivariate normal densities:

$$\begin{aligned}
&\frac{\det\left(2\pi(\Sigma_{11} + W\Sigma_{11}^2)\right)^{-1/2} \exp\left(\frac{1}{2} Z_1^T (\Sigma_{11} + W\Sigma_{11}^2)^{-1} Z_1\right)}{\det(2\pi\Sigma_{11})^{-1/2} \exp\left(\frac{1}{2} Z_1^T (\Sigma_{11})^{-1} Z_1\right)} \\
&= \det\left(\Sigma_{11}^{-1}(\Sigma_{11} + W\Sigma_{11}^2)\right)^{-1/2} \exp\left(\frac{1}{2} Z_1^T \left(\Sigma_{11}^{-1} - (\Sigma_{11} + W\Sigma_{11}^2)^{-1}\right) Z_1\right)
\end{aligned} \tag{10}$$

It remains to show that $\Sigma_{11}^{-1} - (\Sigma_{11} + W\Sigma_{11}^2)^{-1} = W(I + W\Sigma_{11})^{-1}$. This is an immediate consequence of the "matrix inversion lemma":

$$A^{-1} + B^{-1} = A^{-1}(A + B)B^{-1}, \tag{11}$$

using $A = \Sigma_{11}$ and $B = -(\Sigma_{11} + W\Sigma_{11}^2)$.

The easiest way to prove Lemma 1 is to apply our strategy from Lemma 2 to both sides.

5 GWAS/LD mismatch

For simplicity, assume that there are only two variants and denote:

$$Z = \begin{bmatrix} a \\ b \end{bmatrix}, \qquad \Sigma = \begin{bmatrix} 1 & r \\ r & 1 \end{bmatrix}. \tag{12}$$

Let BF_a, BF_b, BF_{ab} be the Bayes factors for configurations where the first variant, the second variant or both are causal, respectively. In configurations with a single causal variant, the causal block $\Sigma_{11} = \begin{bmatrix} 1 \end{bmatrix}$; in particular, the Bayes factor is independent of the correlation r. According to Lemma 2:

$$\begin{aligned}
BF_a &= (1 + W)^{-1/2} \exp\left(\frac{W}{2(1 + W)} a^2\right), \\
BF_b &= (1 + W)^{-1/2} \exp\left(\frac{W}{2(1 + W)} b^2\right).
\end{aligned} \tag{13}$$

(As a side note, replacing W by W/V, where V is the variance in the estimate of the regression coefficient, gives the Bayes factors from coloc. So these models are closely related.)

For the configuration with two causal variants, the causal block $\Sigma_{11} = \Sigma$, so:

$$\det(I+W\Sigma_{11}) = (1+W)^2 - W^2r^2, \qquad (I+W\Sigma_{11})^{-1} = \frac{1}{(1+W)^2 - W^2r^2}\begin{bmatrix} 1+W & -Wr \\ -Wr & 1+W \end{bmatrix}. \tag{14}$$

$$BF_{ab} = \left((1+W)^2 - W^2r^2\right)^{-1/2} \exp\left(\frac{W}{2((1+W)^2 - W^2r^2)}\left[(1+W)(a^2+b^2) - 2Wrab\right]\right) \tag{15}$$

In the case r=0, 15 reduces to:

$$BF_{ab}^{r=0} = (1+W)^{-1}\exp\left(\frac{W}{2(1+W)}(a^2+b^2)\right). \tag{16}$$

So:

$$\frac{BF_{ab}^{r=0}}{BF_a} = (1+W)^{-1/2}\exp\left(\frac{W}{2(1+W)}b^2\right). \tag{17}$$

- In other words, when the two variants are uncorrelated, the likelihood ratio of C_{ab} relative to C_a increases exponentially with b. This makes sense, because b is bringing new information that cannot be explained by a alone.
- To have significant posterior probability mass on C_{ab}, the exponential factor must compete with $(1+W)^{-1/2}$, as well as with the prior, which gets smaller with the inclusion of more causal variants.
- For this to work well in practice, W must not be too small; say $W > 1$.

In the case r=1, 15 reduces to:

$$BF_{ab}^{r=1} = (1+2W)^{-1/2}\exp\left(\frac{W}{2(1+2W)}\left[(a^2+b^2) + W(a-b)^2\right]\right), \tag{18}$$

so:

$$\frac{BF_{ab}^{r=1}}{BF_a} = \left(\frac{1+2W}{1+W}\right)^{-1/2}\exp\left(\frac{W}{2(1+2W)}\left[b^2 - \frac{W}{1+W}a^2 + W(a-b)^2\right]\right). \tag{19}$$

Since $r = 1$, we would expect that $b \simeq a$, in which case $W(a-b)^2 \simeq 0$. If W is large enough, then $b^2 - \frac{W}{1+W}a^2$ is also small, and the exponent does not grow too fast. In this case, C_a and C_b end up with more probability mass than C_{ab}: the model correctly infers that either variant can be causal, but both are unlikely.

However, if LD is obtained from a reference panel which does not match the GWAS population well, it is possible to have $r = 1$ and $b \not\simeq a$. In this case, as $W(a-b)^2$ becomes large, $BF_{ab}^{r=1}$ dominates not only BF_a and BF_b, but also the Bayes factors for variants other than the two considered here. In practice, we even see examples where $r = 1$, but a and b have different signs. In these cases, most of the probability mass ends up on the configuration C_{ab}, at the expense of other variants which have stronger GWAS association than either a or b!

In practice, we can mitigate this danger by not letting W be too large, thus controlling the term $W(a-b)^2$; say $W < 20$. Together with the earlier requirement, we find that a reasonable range is $1 < W < 20$.

Methods References

[1] Gleb Kichaev, Wen-Yun Yang, Sara Lindstrom, Farhad Hormozdiari, Eleazar Eskin, Alkes L. Price, Peter Kraft, and Bogdan Pasaniuc. Integrating functional data to prioritize causal variants in statistical fine-mapping studies. *PLOS Genetics*, 10(10):1–16, 10 2014.

[2] Farhad Hormozdiari, Martijn van de Bunt, Ayellet V. Segrè, Xiao Li, Jong Wha J. Joo, Michael Bilow, Jae Hoon Sul, Sriram Sankararaman, Bogdan Pasaniuc, and Eleazar Eskin. Colocalization of gwas and eqtl signals detects target genes. *The American Journal of Human Genetics*, 99(6):1245–1260, 2016.
